# C1019T Polymorphism in the Connexin 37 Gene and Myocardial Infarction Risk in Premature Coronary Artery Disease 

**Published:** 2017-04

**Authors:** Mehrdad Sheikhvatan, Mohammadali Boroumand, Mehrdad Behmanesh, Seyed Hesameddin Abbasi, Gholamreza Davoodi, Shayan Ziaee, Sara Cheraghi

**Affiliations:** 1 *Tehran Heart Center, Tehran University of Medical Sciences, Tehran, Iran.*; 2 *Department of Human Genetics, Tarbiat Modarres University, Tehran, Iran.*

**Keywords:** *Genes*, *Connexin 37*, *Myocardial infarction*, *Polymorphism, genetic*

## Abstract

**Background:** The C1019T polymorphism of the connexin-37 (GJA4) gene is a single-nucleotide polymorphisms involved in atherosclerotic plaque rupture and atherosclerosis predisposition. We examined the association between the C1019T polymorphism of the GJA4 gene and the occurrence of myocardial infarction (MI) in patients with premature coronary artery disease (CAD).

**Methods:** Our study recruited 1000 patients with the final diagnosis of premature CAD and classified them into 2 groups: with a history of MI (n = 461) and without it (n = 539). The polymorphism variants were determined via the PCR–RFLP, and then genotyping was conducted through the high-resolution melting method. From a total of 1000 patients, 554 patients, who had been previously followed-up with a median follow-up time of 45.74 months vis-à-vis long-term major adverse cardiac events, were enrolled in this retrospective cohort phase.

**Results:** The frequencies of the wild, heterozygous, and mutant genotypes of the C1019T polymorphism were 54.0%, 40.6%, and 5.4% in the MI group and 49.2%, 43.2%, and 7.6% in the non-MI group (p value = 0.187). After adjustment for the baseline covariates, no difference was found between the MI and non-MI groups apropos the frequency of the heterozygous genotype (p value = 0.625) and the mutant genotype (p value = 0.452). Regarding the level of human connexin-37, the serum level of this marker was not different between the MI and non-MI groups.

**Conclusion:** The C1019T polymorphism of the GJA4 gene may not be useful for predicting the occurrence of MI in patients with premature CAD. The presence of this polymorphism in such patients may also have a low value for predicting long-term CAD complications.

## Introduction

Early-onset myocardial infarction (MI) is a major cause of death and disability and occurs as a life-threatening consequence of coronary artery disease (CAD) in the young population. The occurrence of MI among young individuals, especially in developed nations, is of utmost importance because of the heavy financial burden it imposes on the community. According to a recent report, although the majority of MI cases are observed in individuals > 65 years old, about 5%–10% of new MI cases occur in younger patients.^1^^, ^^2^ Interestingly, the occurrence of MI in young individuals is associated with greater heritability than that in the elderly. Some genome-wide association studies have been able to identify several single-nucleotide polymorphisms (SNPs) that are reproducibly associated with the risk of new MI in the young population.^3^ Although familial aggregation in the occurrence of new MI in young individuals has been revealed, its pathophysiological nature and also the population-based gene polymorphisms that contribute to its occurrence have remained uncertain.^4^ Nevertheless, what has been clearly determined is the essential role of family history of cardiac ischemic events in the occurrence of MI in young patients with proven CAD.^5^

The main fundament of MI is atherosclerotic plaque rupture, which occurs in consequence of shear forces in the artery, gradual decay, or mechanical injuries. Plaque rupture can induce activation, adhesion, and aggregation in ruptured plaques by expressing some specific platelet surface receptors and also by activating some receptors in the position of ruptured plaques. The activation of these receptors may result in thrombus formation, clot development, and finally limitation of the coronary blood flow and lead to myocardial ischemia. The activity of platelet surface receptors and the receptors on the endothelial layer is under the direction and control of various genes. In this context, the occurrence of polymorphisms in these genes may be associated with behavioral changes of the gene in the excitation or prevention of ischemic events. 

One of the identified SNPs involved in atherosclerotic plaque rupture is the C1019T polymorphism of the connexin-37 (GJA4) gene.^6^^, ^^7^ The GJA4 gene encodes a set of proteins called “connexins”, which play an important role in regulating and structuring intercellular channels. In other words, the passage of low-weight molecules through the intercellular channels and their movement from one cell to another is mainly regulated by the activation of this gene, and this role has also been proven in atherosclerotic plaques.^8^^-^^10^ Consequently, it has been posited that the occurrence of the C1019T polymorphism is accompanied by the instability of plaques and the occurrence of acute MI.^11^ However, only a few studies have so far assessed the role of this polymorphism as a trigger for early-onset MI, especially in young ages. Hence, we sought to examine the association between the C1019T polymorphism of the GJA4 gene and the occurrence of MI in Iranian patients with premature CAD.

## Methods

One thousand patients, comprising 492 men and 508 women, at a mean age of 45.51 ± 5.88 years (age = 21–55 y), were prospectively enrolled into the Tehran Heart Center Cardiovascular Epigenetic Cohort Study (THC-CEC) from 2008 to 2014. THC is a major referral hospital for patients with CAD from around the country. Each participant in the present study had a confirmed diagnosis of premature CAD, defined as the occurrence of the disease before 45 years old in men and before 55 years old in women. Angiographically identified stenoses > 50% in 1 of the major coronary vessels at the time of the study were used to classify the patients as having single-vessel, double-vessel, or triple-vessel disease. According to clinical symptoms (typical chest discomfort), specific electrocardiographic (ECG) changes, and elevation of cardiac enzymes (creatine kinase-MB [CK-MB] and troponin T), the enrolled patients were classified as the MI group (n = 461) and the non-MI group (n = 539).^12^ All the subjects had CAD, but those with evidence of MI were classified as the MI group and those with no evidence of MI were classified as the non-MI group. The participants were prospectively interviewed as early as possible during their admission to ascertain their demographic characteristics, medical history, and medication. The results of laboratory tests and disease severity based on coronary angiography were collected by reviewing the THC Angiography Database. Approval from the Research Review Board and Ethics Committee of Tehran University of Medical Sciences was obtained before the commencement of the study, and a written informed consent to participate was signed by each participant. 

The severity of CAD was determined based on the number of involved coronary arteries according to angiography reports. The left ventricular ejection fraction was measured quantitatively via echocardiography, just before angiography, using the Simpson method. With regard to cardiovascular risk factors, current smoking was defined as smoking tobacco product/products once or more times per day or having smoked in a 30-day period prior to admission. Hypercholesterolemia was defined as total cholesterol ≥ 5.0 mmol/L, high-density lipoprotein cholesterol ≤ 1.0 mmol/L in men and ≤ 1.1 mmol/L in women, or triglyceride ≥ 2.0 mmol/L. A positive family history of CAD was defined as the presence of the disease among 1st-degree relatives before the age of 55 in men and 65 years in women. Hypertension was defined as a systolic blood pressure ≥ 140 mmHg and/or a diastolic blood pressure ≥ 90 mmHg and/or receiving antihypertensive treatment. Diabetes mellitus was defined as symptoms of diabetes plus a plasma glucose concentration ≥ 11.1 mmol/L or fasting plasma glucose ≥ 7.0 mmol/L or 2-hp ≥ 11.1 mmol/L.^13^

**Figure 1:**
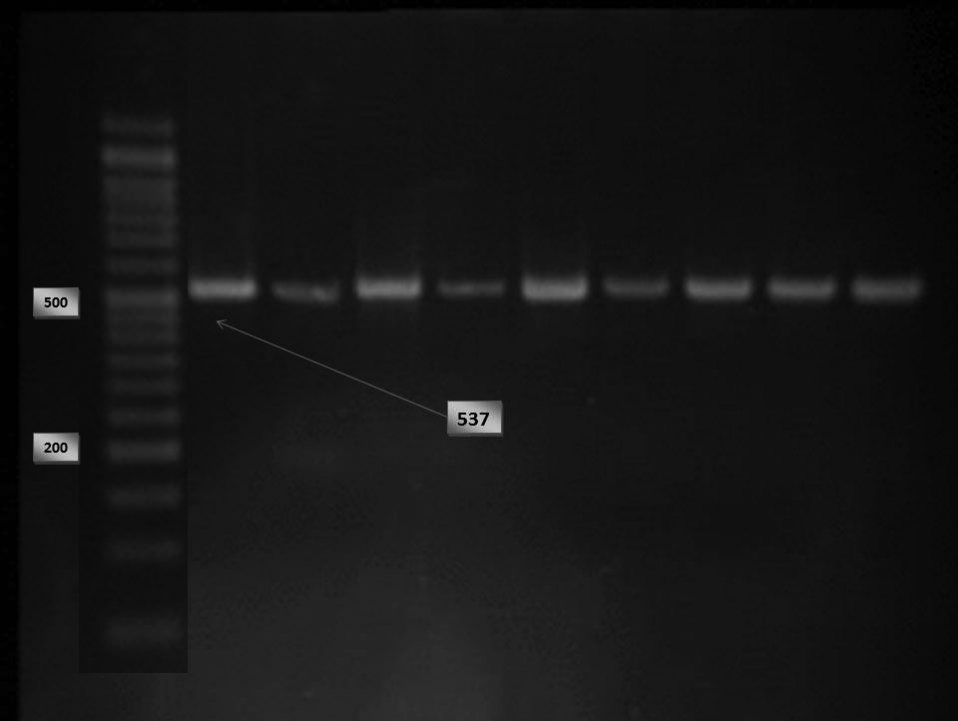
Electrophoresis for presenting the PCR product of the GJA4 gene

**Figure 2:**
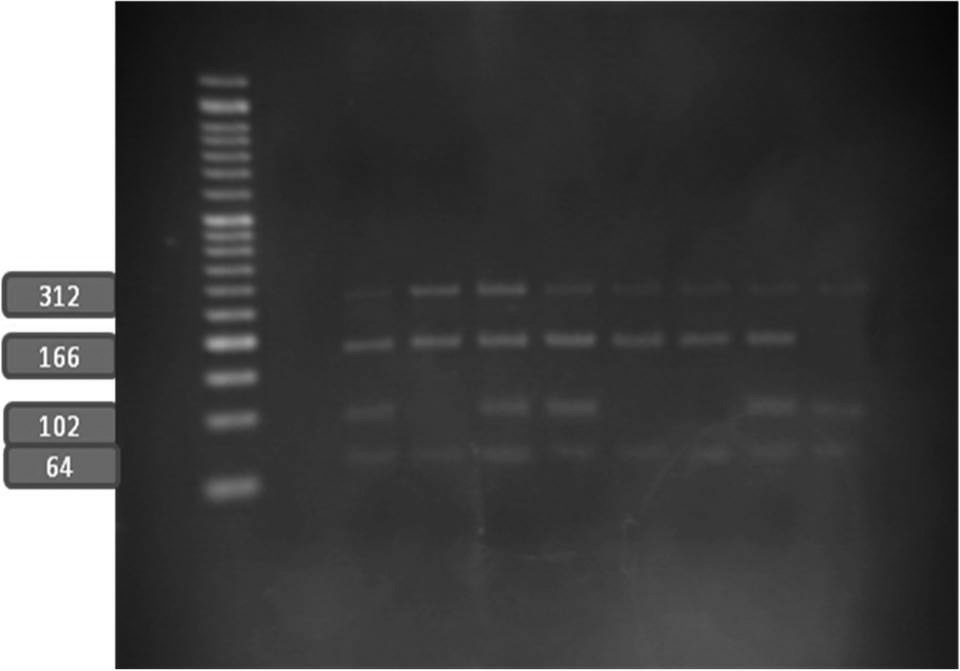
PCR-RFLP analysis with Mae III for the C1019T polymorphism to determine the polymorphism variants

**Figure 3:**
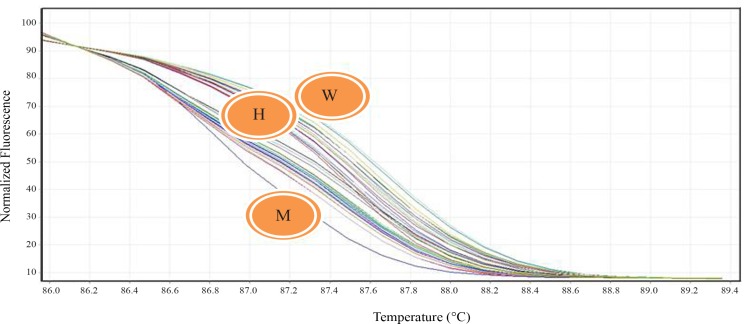
High-resolution melting genotyping of the C1019T polymorphism

Genomic DNA was isolated from peripheral blood leukocytes according to a standard salting‐out method.^12^ Briefly, buffy coats of nucleated cells obtained from anticoagulated blood were resuspended in polypropylene centrifugation tubes with a nuclei lysis buffer. The cell lysates were digested overnight at 37 °C with 10% Sodium Dodecyl sulphate (SDS) and a protease K solution. After the completion of digestion, saturated NaCl was added to the tubes and the tubes were shaken vigorously. Afterward, centrifugation was performed. The precipitated protein pellet was left at the bottom of the tubes, and the supernatant containing the DNA was transferred to another tube. Absolute ethanol was then added, and the tubes were inverted several times until the DNA precipitated. The precipitated DNA strands were removed and transferred to a microcentrifuge tube. The DNA was allowed to dissolve at 37 °C before quantitative analysis. The polymorphisms were genotyped using the polymerase chain reaction–restriction fragment length polymorphism method (PCR–RFLP ) with a 25-μL reaction mixture containing 0.6 μL of DNA, 0.6 μM of each primer, and 12.5 μM of Taq PCR Master Mix (Qiagen, Valencia, CA) and employing primers forward 5’-TGGACCCACCCCCTCAGAATGGCCAAAGA 3’ and reverse 5’ AGGAAGCCGTAGTGCCTGGTGG 3’ digested by restriction enzyme Mae III. The digested products were subsequently visualized on 3% agarose gel stained with ethidium bromide (Figure 1 and Figure 2). To final draft the determined genotypes of the SNPs in RFLP, we performed DNA sequencing in some samples of the different genotypes in the SNP and to determine the genotype patterns of the C1019T polymorphism of the GJA4 gene in all the enrolled subjects, we applied the high-resolution melting (HRM) technique using specific primers forward 5’- CAACCTGACCACAGAGGAGAG -3’ and reverse 5’- CTTAGAAGCAGAGCTGCTGG -3’ with a Rotor-Gene 6000 (Corbett Life Science) (Figure 3). The relationship between the polymorphism and the serum level of the gene product was examined via the measurement of the serum level of Human Gap junction alpha-4 protein using an ELISA kit (# CSB-EL009447HU, Wuhan Hi-tech, China). 

The 537-bp bands were detected by 2% agarose gel electrophoresis following the PCR amplification of the GJA4 gene sequences.

PCR, Polymerase-chain-reaction

Agarose gel shows the bands obtained by the electrophoresis of the RFLP products of the PCR products of the GJA4 gene using restriction enzyme Mae III.

PCR-RFLP, Polymerase chain reaction – Restriction fragment length polymorphism

The genotype patterns of the C1019T polymorphism of the GJA4 gene are shown as wild (W), heterozygous (H), and mutant (M) in the enrolled subjects.

**Figure 4:**
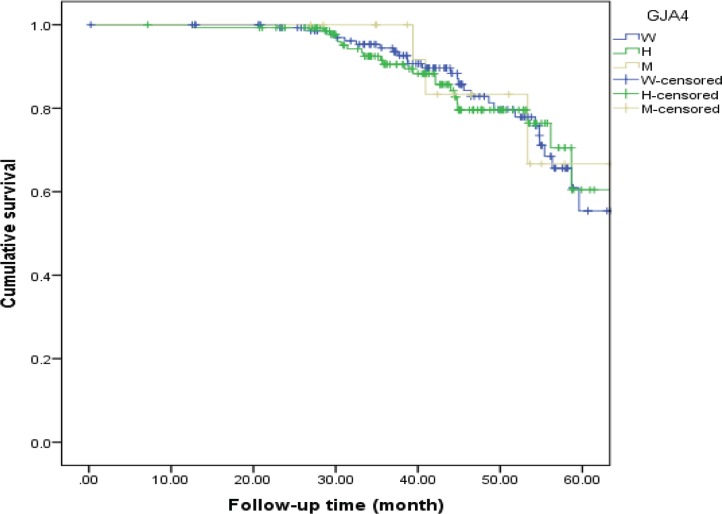
MACE-free Kaplan–Meier survival curve in the non-MI patients

MACE, Major adverse cardiac events; MI, Myocardial infarction

**Figure 5:**
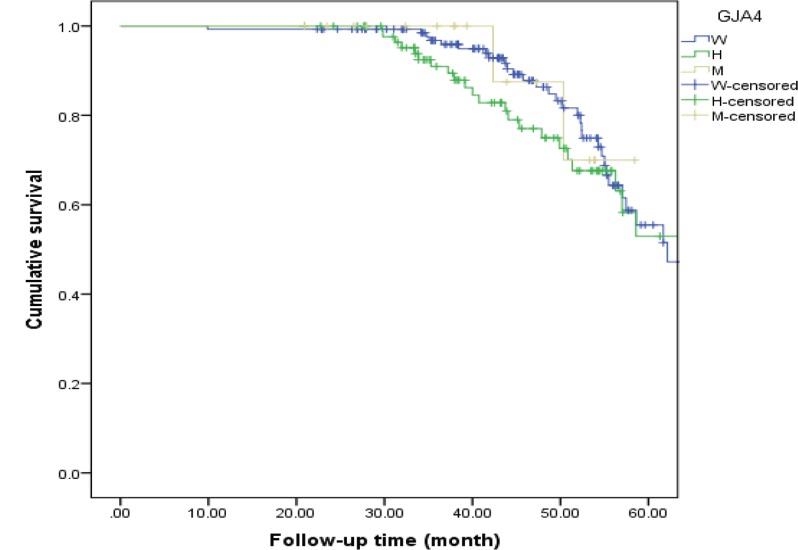
MACE-free Kaplan–Meier survival curve in the MI patients

MACE, Major adverse cardiac events; MI, Myocardial infarction

In this phase, the predictive value of the C1019T polymorphism for CAD-related complications was assessed. Out of the initial 1000 patients, a total of 554 patients, who had been previously followed-up with a median follow-up time of 45.74 months apropos long-term major adverse cardiac events (MACE), were enrolled in this retrospective cohort phase. An assessment of the Hardy–Weinberg equilibrium in the 554 patients showed the restoration of the equilibrium (p value = 0.490). Total MACE was defined as the presence of at least 1 of the following cardiovascular outcomes: new coronary involvement (defined as a new diseased coronary vessel or expansion of the previous coronary involvement based on angiography), new MI, undergoing coronary artery bypass surgery or percutaneous coronary intervention, and brain stroke. The study end point was to determine the total MACE-free survival rate in the 2 groups of with and without MI and also to assess the value of the C1019T polymorphism in predicting total MACE in both MI and non-MI groups. 

The quantitative variables are presented as means ± standard deviations (SDs) (for the variables with a normal distribution) or median (1st and 3rd quartiles) (for the variables without a normal distribution), and the categorical variables are presented as absolute frequencies and percentages. The continuous variables were compared using the *t*-test. The Mann–Whitney *U*-test was used when the data did not meet the assumptions of the parametric test, especially the assumption about the normally distributed data. The categorical variables were compared using the χ^2^ test. The differences in the frequencies of the different genotypic patterns between the MI and non-MI groups were examined unadjusted and then adjusted for the baseline characteristics and the clinical data using multiple logistic regression modeling. Variables with a p value < 0.2 in the univariate analysis were included in the multivariable model. The Cox proportional hazard model was employed to analyze the association between the existence of gene polymorphism and total MACE in both MI and non-MI groups and the hazard ratio (HR) was then displayed. Postoperative survival was assessed using the Kaplan–Mayer curve. A p value ≤ 0.05 was considered statistically significant. The data were analyzed using IBM SPSS, version 21.0 (Armonk, NY: IBM Corp.).

## Results

A comparison of the MI and non-MI groups with respect to their baseline variables (Table 1) showed a lower mean age (43.78 ± 5.61 y vs. 47.00 ± 5.70 y; p value = 0.004), higher frequency of male gender (66.2% vs. 34.7%; p value < 0.001), and lower mean body mass index (29.06 ± 4.71 kg/m^2^ vs. 30.26 ± 5.36 kg/m^2^; p value = 0.017) in the MI group. Regarding the cardiovascular risk profile, although no significant difference was indicated in terms of family history of CAD between the 2 groups (33.8% vs. 36.2%; p value = 0.440), the differences between the groups in the other underlying risk factors were meaningful (Table 1). The MI group also had a lower mean left ventricular ejection fraction as well as higher diseased coronary vessels. 

The distribution of the genotypes was not significantly different according to the Hardy–Weinberg equilibrium between the MI group (χ^2^ = 0.40) and the non-MI group (χ^2^ = 0.20). The frequencies of the wild, heterozygous, and mutant genotypes of the C1019T polymorphism were 54.0%, 40.6%, and 5.4% in the MI group and 49.2%, 43.2%, and 7.6% in the non-MI group (p value = 0.187). There were also no significant differences as regards the polymorphism genotypes between the 2 study groups after adjustment for the baseline covariates, namely the demographic variables, risk factors, oral medication, and severity of coronary artery involvement (adjusted OR = 0.930; p value = 0.625 for the heterozygous genotype, and adjusted OR = 0.777; p value = 0.452 for the mutant genotype) (Table 2). Concerning the level of human β3 integrin, the serum level of this marker was 31.23 ± 35.61 mg/dL in the MI group and 34.77 ± 37.96 mg/dL in the non-MI group, with no significant difference (p value = 0.655). 

The MI and non-MI groups were followed-up for median times of 44.55 months and 46.92 months, respectively, to assess the differences in the total MACE-free survival rate across the different genotypes of the C1019T polymorphism. In the non-MI group, MACE occurred in 19.3% of the patients with the wild genotype, in 16.2% with the heterozygous genotype, and in 17.6% with the mutant genotype. In this regard, the 4-year MACE-free survival rates in the patients with wild, heterozygous, and mutant patterns were 82.8%, 79.6%, and 83.3%, correspondingly. Also, in the MI group, MACE occurred in 23.2% of the patients with the wild genotype, in 27.5% with the heterozygous genotype, and in 11.7% with the mutant genotype. In this regard, the 4-year MACE-free survival rates in the patients with wild, heterozygous, and mutant patterns were 86.3%, 75.0%, and 70.0%, respectively. In addition, no difference was revealed in the total MACE-free survival rate between the MI patients with the different genotypes of the C1019T polymorphism (HR = 1.432, 95%CI: 0.844–2.428; p value = 0.183 for the heterozygous genotype, and HR = 0.922, 95%CI: 0.219-3.877; p value = 0.912 for the mutant genotype) (Table 3) and also in the non-MI group (HR = 1.120, 95% CI: 0.639-1.963; p value = 0.692 for the heterozygous genotype and HR = 0.990, 95%CI: 0.300–3.272; p value = 0.987 for the mutant genotype) (Table 4).

**Table 1:** Baseline characteristics and clinical data in both MI and non-MI groups

	MI Group (n = 461)	Non-MI Group (n = 539)	P value
Gender			
Male	305 (66.2)	187 (34.7)	< 0.001
Female	156 (33.8)	352 (65.3)	
Age (y)	43.78±5.61	47.00±5.70	0.004
Body mass index (kg/m^2^)	29.06±4.71	30.26±5.36	0.017
Medical history			
Family history of CAD	156 (33.8)	195 (36.2)	0.440
Current smoking	195 (42.3)	102 (18.9)	< 0.001
Hyperlipidemia	310 (67.2)	415 (77.0)	0.001
Hypertension	206 (44.7)	313 (58.1)	< 0.001
Diabetes mellitus	119 (25.8)	208 (38.6)	< 0.001
Opium use	102 (22.1)	46 (8.5)	< 0.001
Oral medication			
Aspirin	437 (94.8)	467 (86.6)	< 0.001
Beta-blockers	412 (89.4)	402 (74.6)	< 0.001
Nitrate	395 (85.7)	376 (69.8)	< 0.001
Calcium blocker	51 (11.1)	92 (17.1)	0.007
Antihyperlipidemic	30 (6.5)	31 (5.8)	0.618
Antihyperglycemic	71 (15.4)	136 (25.2)	< 0.001
Digoxin	10 (2.2)	8 (1.5)	0.417
Diuretics	37 (8.0)	53 (9.8)	0.320
ACE-inhibitor	312 (67.7)	238 (44.2)	< 0.001
Number of involved coronary arteries			
One vessel	221 (47.9)	293 (54.4)	0.005
Two vessels	107 (23.2)	136 (25.2)	
Three vessels	133 (28.9)	110 (20.4)	
Left ventricular ejection fraction (%)	46.52±10.14	55.58±7.98	< 0.001
Total cholesterol (mg/dL)	182.92±53.81	193.82±49.25	0.891
Triglyceride (mg/dL)	170.0 (132.0–228.5)	160.0 (117.5–223.0)	0.040
Low-density lipoprotein (mg/dL)	106.0 (84.0–131.5)	118.0 (91.0–149.0)	< 0.001
High-density lipoprotein (mg/dL)	38.04±10.23	42.68±11.21	0.047
Fasting blood sugar (mg/dL)	101.0 (90.0–122.0)	104.0 (93.0–149.0)	0.003
Creatinine (mg/dL)	0.9 (0.8–1.1)	0.8 (0.7–1.0)	< 0.001

The quantitative data are presented as means ± standard deviations or medians (1st and 3rd quartiles), and the categorical variables are presented as numbers (%).

The quantitative data were compared using the *t*-test or the Mann–Whitney *U*-test (if nonparametric), and the categorical variables were compared using the χ^2 ^test.

MI, Myocardial infarction; CAD, Coronary artery disease; ACE, Angiotensin-converting enzyme

**Table 2:** Univariate and multivariable logistic regression analyses to determine the association between the C1019T polymorphism and the occurrence of MI adjusted for confounders

	Univariate Analysis		Multivariable Analysis	
	P value	Odds ratio (95% CI)	P value	Odds ratio (95% CI)
C1019T polymorphism				
Wild genotype	0.187	1.000	0.727	1.000
Heterozygous genotype	0.072	0.876 (0.554 – 1.114)	0.625	0.930 (0.670 – 1.292)
Mutant genotype	0.144	0.111 (0.076 – 0.223)	0.452	0.777 (0.402 – 1.501)
Male gender	< 0.001	3.324 (1.779 – 3.885)	0.006	2.006 (1.224 – 3.287)
Current smoking	< 0.001	1.895 (1.224 – 2.446)	0.025	1.558 (1.058 – 2.294)
Hyperlipidemia	0.001	0.776 (0.256 – 1.149)	0.247	0.806 (0.558 – 1.162)
Hypertension	< 0.001	1.456 (1.224 – 2.746)	0.644	1.086 (0.764 – 1.545)
Diabetes mellitus	< 0.001	0.568 (0.225 – 0.879)	0.267	0.804 (0.548 – 1.181)
Aspirin use	< 0.001	1.897 (1.789 – 2.453)	0.509	1.257 (0.637 – 2.482)
Beta-blocker use	< 0.001	4.526 (1.752 – 5.789)	< 0.001	3.050 (1.805 – 5.155)
Nitrate use	< 0.001	2.254 (1.014 – 3.145)	0.010	1.793 (1.151 – 2.793)
Calcium use	0.007	0.878 (0.779 – 0.986)	0.691	0.912 (0.579 – 1.436)
Three-vessel disease	0.005	1.789 (1.112 – 2.789)	0.050	1.476 (0.999 – 2.179)
Age	0.004	0.456 (0.224 – 659)	0.052	0.961 (0.923 – 1.000)
Body mass index	0.017	1.456 (1.214 – 2.478)	0.736	1.006 (0.973 – 1.040)
Left ventricular ejection fraction	< 0.001	0.789 (0.478 – 0.925)	< 0.001	0.893 (0.876 – 0.910)
Serum creatinine	< 0.001	1.789 (1.256 – 2.145)	0.126	1.385 (0.912 – 2.101)

MI, Myocardial infarction

Hosmer–Lemeshow goodness of fit: χ^2 ^= 5.819; p value = 0.561

**Table 3:** Cox proportional hazard model for assessing the relation between the patterns of the C1019T polymorphism and the occurrence of long-term total MACE adjusted for confounders in the MI group

	Univariate Analysis		Multivariable Analysis	
	P value	Hazard ratio (95% CI)	P value	Hazard ratio (95% CI)
GJA4 (W)	0.360	1.000	0.304	1.000
GJA4(H)	0.224	1.779 (0.113-2.145)	0.183	1.432 (0.844-2.428)
GJA4(M)	0.812	0.877 (0.455-1.256)	0.912	0.922 (0.219-3.877)
Hypertension	0.180	0.884 (0.589-1.256)	0.300	0.730 (0.402-1.324)
Diabetes mellitus	0.037	1.655 (1.478-2.123)	0.824	0.924 (0.460-1.855)
Diuretic use	0.002	1.789 (1.256-2.786)	0.538	0.743 (0.289-1.911)
Serum low-density lipoprotein	0.167	0.789 (0.256-1.576)	0.765	1.001 (0.993-1.009)
Serum creatinine	0.116	0.879 (0.478-1.244)	0.745	0.863 (0.354-2.100)

MACE, Major adverse cardiac events; MI, Myocardial infarction

**Table 4:** Cox proportional hazard model for assessing the relation between the patterns of the C1019T polymorphism and the occurrence of long-term total MACE adjusted for confounders in the non-MI group

	Univariate Analysis		Multivariable Analysis	
	P value	Hazard ratio (95% CI)	P value	Hazard ratio (95% CI)
GJA4 (W)	0.500	1.000	0.790	1.000
GJA4(H)	0.570	1.789 (0.555-1.879)	0.692	1.120 (0.639-1.963)
GJA4(M)	0.788	0.811 (0.455-2.998)	0.987	0.990 (0.300-3.272)
Diuretic use	0.035	0.566 (0.179-0.795)	0.021	0.412 (0.194-0.873)
Serum FBS	0.032	1.012 (1.004-1.056)	0.003	1.006 (1.002-1.010)

MACE, Major adverse cardiac events; MI, Myocardial infarction; FBS, Fasting blood sugar

## Discussion

The current study attempted to determine the association between the C1019T polymorphism, located in the GJA4 gene, and the occurrence of MI in patients with premature CAD. In the next step, we sought to assess the value of this polymorphism in predicting long-term MACE in patients with premature CAD. We succeeded in showing that this polymorphism could not predispose patients with premature CAD to MI, and nor could it predict long-term cardiovascular events in these patients. In this context, we focused on a large sample of the Iranian population with premature CAD, who referred to our hospital as a main referral center for managing CAD in Iran. Because our sample is an appropriate sample of all races and ethnicities in Iran, the obtained results can be generalized to the entire Iranian population. 

A review of the literature (Table 5) shows that most relevant studies have emphasized the role of this polymorphism in the prediction of an increased risk for MI in diverse communities.^13^^-^^21^ Even in a similar study in our country, the association between the C1019T polymorphism and the occurrence of acute MI was significant. However, in a study on the young Finnish population, the presence of this polymorphism was not associated with the risk for the development of atherosclerosis in that population.^19^ The common denominator of our study population with the Finnish study is the selection of young adults and also the assessment of the risk for early MI. On the other hand, our insignificant results vis-à-vis the association between the C1019T polymorphism and the occurrence of acute MI may be related to the nature of CAD prematurity. This is, however, a crude hypothesis that requires further evaluation. Furthermore, in the present study, we specifically used a new HRM technique for final genotyping. Although this technique is a highly sensitive, simple, and low-cost test to detect human disease-associated mutations, especially for samples with mutations of low incidence, the sensitivity of this technique varies according to the number of samples with/without mutations and, thus, positive results require DNA sequencing for confirmation because its specificity has shown a considerable heterogeneity between different studies.^22^ Another limitation of this technique is that different heterozygotes may produce melting curves so similar to each other that, although they clearly vary from homozygous variants, they are not differentiated from each other.^23^

**Table 5:** Review of the literature on the association between polymorphism and the occurrence of myocardial infarction in patients with coronary artery disease

	Country	Publication	Number of patients	Association between polymorphism and myocardial infarction
Yamada et al.^13^	Japan	2002	2819	Yes
Lanfear et al.^14^	USA	2004	190	Yes
Listı` et al.^15^	Italy	2005	293	Yes
Wong et al.^16^	Switzerland	2007	781	Yes
Collings et al.^17^	Finland	2007	1440	No
Seifi et al.^18^	Iran	2013	385	Yes
Balatskiĭ et al.^19^	Russia	2013	183	Yes
Our study	Iran	2014	1000	No

Connexin-37 is a gap junction protein encoded by the GJA4 gene and is induced in the vascular smooth muscle during coronary arteriogenesis. It has been well demonstrated that the expression of this protein can be altered in atherosclerotic lesions in both human and animal models. In fact, because normal connexin-37 can inhibit leukocyte adhesion mediated by the release of ATP into the extracellular space, the anti-adhesive effect can be altered in those with the GJA4 gene polymorphisms predisposing to leukocyte adhesion.^24^ Furthermore, connexin-37 has a central role as an intrinsic negative regulator of platelet function, so that its deficiency following the GJA4 gene mutations may result in platelet aggregation and lead to thrombus growth. In sum, these mechanisms due to the GJA4 gene polymorphisms may help predict atherosclerosis complications such as MI.^25^^, ^^26^ However, in our observation, this role for the C1019T polymorphism of the GJA4 gene was not demonstrated. This result may have been due to our selecting a young CAD population in our survey. It seems that the development and localization of connexin proteins to the gap junctional spots may be age-dependent as is shown in some animal studies^27^ and, accordingly, their related polymorphisms may be phenotypic by increasing age. This new point should be examined by employing 2 young and older age subgroups of CAD patients and comparing the GJA4 gene polymorphism state between these groups.

As a limitation in our study, the data obtained from a previous cohort study, which followed up 554 out of 1000 patients, were merged with our findings so as to determine the association between the genotypes and the patients’ long-term outcome: This might have led to selection bias. The reason was that our study was part of a large study in THC on both epidemiological and genetic aspects of premature CAD and, thus, we used the follow-up findings obtained by the parallel epidemiological study on the same population. 

## Conclusion

The C1019T polymorphism of the GJA4 gene may not be useful for predicting the occurrence of MI in patients with premature CAD. Also, the presence of this polymorphism in this group of patients may have a low value for predicting long-term CAD complications. Because of the probable age-related changes in the functions of the connexin gap junction protein, the predictive role of this polymorphism may be observable in the old population, not in patients with premature CAD. This topic merits further assessment in future association studies. 

## Notes:

***This paper should be cited as:*** Sheikhvatan M, Boroumand M, Behmanesh M, Abbasi SH, Davoodi G, Ziaee S, Cheraghi S. C1019T Polymorphism in the Connexin 37 Gene and the Risk for Myocardial Infarction in Premature Coronary Artery Disease. J Teh Univ Heart Ctr 2017;12(2):72-81.
